# Lack of Skin of Color Representation in Dermatology-Related Instagram Posts: Content Analysis

**DOI:** 10.2196/37415

**Published:** 2022-05-24

**Authors:** Fahad Ahmed, Princess Ogidi, Omar Shareef, Jules Lipoff

**Affiliations:** 1 Department of Dermatology Perelman School of Medicine University of Pennsylvania Philadelphia, PA United States; 2 Drexel College of Medicine Drexel University Philadelphia, PA United States; 3 Harvard John A Paulson School of Engineering and Applied Sciences Harvard College Cambridge, MA United States

**Keywords:** skin of color, Instagram, dermatology, eHealth, skin photographs, social media, skin condition, skin, health information, skin care, content, information, representation, photo, posts

## Introduction

There is growing recognition that disparities in health care utilization affect patient outcomes. Notably, Black and Hispanic patients are more than 45% less likely compared to White patients to utilize dermatology care for a skin condition [1]. One factor contributing to health care underutilization by racial and ethnic minority groups may be the lack of health information [2]. Previous work has studied the credentials of social media influencers in dermatology and the lack of skin type diversity in dermatology textbooks [3,4]. Given the increasing use of social media as a health information resource, we aimed to evaluate and characterize skin type representation in popular dermatology-related posts on Instagram [5].

## Methods

Some of the most used Instagram hashtags encompassing common dermatologic diagnoses (#acne, #eczema, #psoriasis), procedures (#botox, #chemicalpeel, #mohs), and #dermatology [6] were selected for review. With every hashtag, Instagram’s “Top Posts” feature was used to account for user-specific feed differences and was evaluated every other day for 15 consecutive days in February 2021. For each post, we recorded account type, number of account followers, engagement rate (ER), and skin type of the featured individual. The social media ER, determined using SocialBlade (a social media analytics website [7]), is a quantitative measure of the amount of interaction that content receives relative to a user’s audience size. Two independent observers (FA and PO) estimated the Fitzpatrick skin type (I-VI). With any ambiguity regarding skin types or when multiple individuals were displayed, photographs were labeled with the highest evident Fitzpatrick score. In cases of interrater disagreement, a third observer (JL) independently evaluated the photograph to reach a consensus. This study was considered exempt by the Institutional Review Board.

## Results

Of the posts reviewed (N=441), 46 (10.4%) displayed skin of color (SOC), characterized as Fitzpatrick types IV to VI (Table 1). Cohen κ, measuring interrater reliability for Fitzpatrick skin type, was almost perfect at 0.87. The mean follower count and ER for lighter skin type (Fitzpatrick types I-III) posts were 167,660 and 3.75%, respectively. The mean follower count and ER for SOC posts were 87,440 and 4.68%, respectively. Of the posts made by provider accounts, 9.8% (17/173) displayed SOC individuals. Counts of Fitzpatrick skin types by hashtag are reported in Table 2.

**Table 1:** Fitzpatrick skin types by account type.

Fitzpatrick skin type	Business accounts (n=128), n	Provider accounts (n=173), n	Personal accounts (n=140), n	Total (N=441), n (%)
I	7	7	4	18 (4.1)
II	78	121	102	301 (68.3)
III	25	28	23	76 (17.2)
IV	10	14	9	33 (7.5)
V	7	3	2	12 (2.7)
VI	1	0	0	1 (0.2)

**Table 2:** Fitzpatrick skin types by hashtag.

Hashtag	Fitzpatrick skin type I-III (n=395), n	Fitzpatrick skin type IV-VI (n=46), n
#acne	57	6
#eczema	56	7
#psoriasis	57	6
#botox^a^	62	1
#chemicalpeel	47	16
#mohs^a^	62	1

^a^The hashtags #botox and #mohs each had the fewest posts (n=1) with darker skin color, possibly due to skin cancer incidence and Botox use being different among the 2 groups.

## Discussion

Our findings suggest that SOC individuals may be underrepresented on dermatology-related Instagram posts and have a smaller reach as demonstrated by lower follower counts. However, SOC posts had a higher ER, suggesting that users were more likely to interact and engage with SOC content. Limitations include Fitzpatrick skin type estimation being based on photographs alone; however, by recording intermediate skin types as darker during data collection, this would have biased against the hypothesis of underrepresentation of SOC. Additionally, the user demographics of Instagram are not publicly available to assess relative underrepresentation. Lastly, this classification system is not a direct proxy of race, and racial and ethnic minority groups may not only have Fitzpatrick skin types IV to VI.

Given the increasing importance of social media in sharing health information, it is imperative that we understand and proactively address the issue of underrepresentation. For example, at one institution, educational physician-created social media videos helped increase health care appointment demand and patient health education [5]. Increasing the number of SOC influencers may also help, similar to how patient-provider racial concordance is correlated with increased trust [2]. Thus, increasing representation in dermatologic content on social media may help achieve an opportunity for improved community outreach for racial and ethnic minority groups. Providers, professional organizations, and commercial organizations can play an active role in improving this representation.

## Abbreviations

ER: engagement rate
SOC: skin of color
